# MBC PathNet: integration and visualization of networks connecting functionally related pathways predicted from transcriptomic and proteomic datasets

**DOI:** 10.1093/bioadv/vbaf197

**Published:** 2025-08-18

**Authors:** Jens Hansen, Ravi Iyengar

**Affiliations:** Mount Sinai Institute for Systems Biomedicine, Icahn School of Medicine at Mount Sinai, New York, NY 10029, United States; Department of Pharmacological Sciences, Icahn School of Medicine at Mount Sinai, New York, NY 10029, United States; Mount Sinai Institute for Systems Biomedicine, Icahn School of Medicine at Mount Sinai, New York, NY 10029, United States; Department of Pharmacological Sciences, Icahn School of Medicine at Mount Sinai, New York, NY 10029, United States

## Abstract

**Motivation:**

Advances in high-throughput technologies have shifted the focus from bulk to single cell or spatial transcriptomic and proteomic analysis of tissues and cell cultures. The resulting increase in gene and/or protein lists leads to the subsequent growth of up- and downregulated pathways lists. This trend creates the need for pathway-network based integration strategies that allow quick exploration of shared and distinct mechanisms across datasets.

**Results:**

Here, we present Molecular Biology of the Cell (MBC) Pathway Networks (PathNet). MBC PathNet allows for quick and easy integration and visualization of networks of functionally related pathways predicted from gene and protein lists using the Molecular Biology of the Cell Ontology and other ontologies. Within networks of hierarchical parent-child relationships or functional relationships, pathways are visualized as pie charts where each slice represents a dataset that predicted that pathway. Sizes of pies and slices can be selected to represent statistical significance or other quantitative measures. In addition, MBC PathNet can generate bar diagrams, heatmaps, and timelines. Fully automated execution from the command line is supported.

**Availability and implementation:**

iyengarlab.org/mbcpathnet; mbc-ontology.org; github.com/SBCNY/Molecular-Biology-of-the-Cell

## 1 Introduction

Whole cell physiological functions are generated by coordinated interactions between functionally related pathways. Each pathway comprises multiple gene products that interact with each other to generate a subcellular activity. High-throughput (HT) technologies such as single cell (sc), nucleus (sn), and spatial transcriptomics or regional proteomics have remarkably increased the opportunities to investigate whole cell physiological dynamics. To reveal mechanistic insight that is not easily identified from initial lists of genes, pathway enrichment analysis allows for hundreds of experimentally obtained gene products to be organized as pathways summarizing functions and activities. Using prior knowledge from selected ontologies, e.g. Gene Ontology (GO) (Ashburner *et al.* 2000, Gene Ontology 2023) and Reactome (Milacic *et al.* 2024), pathway enrichment analysis identifies pathways that are statistically enriched in genes with altered expression. The hierarchical organization of ontologies in parent-child relationships, where the children describe subfunctions of their parents, allows documentation of taxonomical relationships between predicted pathways for the same and different datasets, simplifying the interpretability of the results. To characterize networks of functionally interacting subcellular processes (SCPs) underlying whole cell functions, we have developed the Molecular Biology of the Cell Ontology (MBCO) (Hansen *et al.* 2017). Its annotated hierarchy spans three to four SCP levels where SCPs of the same level describe distinct functions of similar biological details as found in the textbook. Annotated parent-child relationships are enriched with interactions between functionally related SCPs that were inferred from prior knowledge. Consideration of these interactions allows a new enrichment approach, dynamic enrichment analysis, which considers interactions and dependencies between the enriched SCPs.

Multiple programs and websites developed by others allow users to submit gene lists to enrichment analysis. Many of them either implement the right-tailed Fisher’s exact test or the Gene Set Enrichment Analysis algorithm (Subramanian *et al.* 2005). The right-tailed Fisher’s exact test calculates the probability of observing the given or a greater overlap between a user-supplied list of genes and genes annotated to a pathway of interest, assuming both were randomly drawn from a shared background set. This approach depends on the definition of a significance cutoff to determine which genes are included. In contrast, Gene Set Enrichment Analysis (GSEA) analysis ranks all genes by a given metric (e.g. significance) and analyses if genes annotated to a given pathway are enriched towards the top of the ranked list. For *P* value calculation, GSEA repeats the analysis after gene or sample label shuffling.

Many providers of the ontologies, e.g. GO (Ashburner *et al.* 2000, Gene Ontology 2023) and Reactome (Milacic *et al.* 2024), allow enrichment analysis of user-supplied genes on their websites. Additionally, independent platforms allow enrichment analysis with multiple ontologies. EnrichR (Kuleshov *et al.* 2016) is a website enabling enrichment analysis using a comprehensive set of ontologies, gene libraries, and datasets extracted from experimental data. An API allows users to access to EnrichR programmatically (Xie *et al.* 2021). ClusterProfiler is an R package for functional enrichment analysis and results visualization, with a focus on the integrative analysis of multiomics datasets (Wu *et al.* 2021). BlitzGSEA is a Python package that accelerates GSEA by efficiently approximating enrichment score probabilities, allowing rapid analysis of large-scale datasets (Lachmann *et al.* 2022). Among the multiple functionalities offered by the enrichment tools ShinyGo (Ge *et al.* 2020) and Cytoscape (Shannon *et al.* 2003, Ono *et al.* 2025) are the visualization of user-submitted genes in pathway diagrams and protein-protein interaction networks. All these applications are useful in different ways. However, they lack the capability to connect pathways predicted from multiple datasets into networks that underlie whole cell functions.

To combine rapid integration of enrichment results obtained for multidimensional datasets with network-based inference and visualization of functional pathway relationships, we have developed the desktop application Molecular Biology of the Cell (MBC) Pathway Networks (PathNet). The primary output are networks connecting predicted pathways that are functionally or hierarchically related. Pathways are visualized as pie charts where pie slices represent datasets that predicted the pathway with significance. The size of each pie slice can reflect significance levels or other metrics. Gene and protein lists obtained by various software packages analyzing HT experiments can be easily uploaded and subjected to pathway identification. The application implements the right-tailed Fisher’s exact test to allow standard enrichment analysis using MBCO (Hansen *et al.* 2017) GO, (Ashburner *et al.* 2000, Gene Ontology 2023), Reactome (Milacic *et al.* 2024), and two user-supplied ontologies. For MBCO, dynamic enrichment analysis is also supported. Multiple datasets can quickly be combined into groups of interest to enable their integrative analysis within networks of pathways. In addition to pathway networks, results can be shown as bardiagrams, timelines, or heatmaps. Analyzed genes or proteins can be mapped to identified pathways and added to the networks as child nodes. For fully automatic analysis, MBC PathNet can be executed from the command line without any user interface interaction.

## 2 Methods

### 2.1 Implementation and dependencies

MBC PathNet enables enrichment analysis of user-supplied gene and protein lists, using MBCO (Hansen *et al.* 2017) and other ontologies such as GO (Ashburner *et al.* 2000, Gene Ontology 2023) and Reactome (Milacic *et al.* 2024) across 11 different species. MBCO datasets are included with the application. For automatic download of all other datasets, we provide two files containing the download commands for Windows or Linux. Pathway networks generated by the application can be visualized with yED graph editor (yWorks GmbH 2025) or Cytoscape (Shannon *et al.* 2003). Links to the download pages for each graph editor are provided within the same files. Within windows the application can be started by opening the exe-file, within a LINUX environment it can be opened using Mono (mono-project.com), a software platform for the Microsoft NET environment that was sponsored by Microsoft. Terminal commands are provided within the “ReadMe”-file in the main directory. Starting the application from the command line followed by at least one argument, allows automatic data upload, parameter definition and analysis without any additional user interactions. Instructions are also provided in the main folder.

### 2.2 Built-in tours

Multiple built-in tours explaining the key features can be used to gain familiarity with the application. The main menu contains two guidance tours, a regular and a mini tour. The regular tour gives an overview of all main functionalities and each menu panel. To facilitate quick use of the application with minimal time investment the mini tour focuses only on the minimum steps that are needed for data upload and analysis.

### 2.3 Integration groups

A central element of the application is the quick assignment of datasets, i.e. user-supplied lists of genes or proteins, to dataset integration groups. Integration groups should contain those datasets that the user wants to cross-compare. Enrichment results obtained for datasets within the same integration group will be visualized in the same figures. SCP networks predicted for datasets of the same integration group will be merged and cross-connected. Integration groups can either be defined using the functionalities of the application within the “Organize data” menu (see below) or be added to the user-supplied data before upload.

### 2.4 Data upload using the “Gene list” box or “Read data” menu

User-generated datasets can be uploaded into the application, using two different ways. Gene or protein lists can either be copy-pasted into the “Gene list” box or be read automatically from text files, using the “Read data” menu. Official NCBI gene symbols pasted into the “Gene list” box should contain one gene in each row. Before and/or after upload using the “Add dataset” button, the user can specify the name of her/his dataset in the related “Name” text box at the right side of the “Gene list” box. Genes manually uploaded using the “Gene list” box or the “Read data” menu will always be set to capital letters within the application. The “Read data” menu allows quick upload of tens to hundreds of different datasets into the application. Depending on the user’s selection the application will either read a single file or all files within a given directory. The eight text boxes in the menu panel allow specification of column names in user-supplied files that map to selected fields within the application, e.g. the integration group field. Fields with empty text boxes will be ignored during data upload. The application will search each supplied file for specified column names and return an error message, if a name was not found. Files whose names end with “_bgGenes” are assumed to contain a single column of genes or proteins without a headline that will be imported as experimental background genes. The experimental background genes will be automatically mapped to all datasets uploaded from a file with the same name, except the “_bgGenes” ending. Experimental background genes should contain all genes or proteins that were tested for significance, e.g. for being differentially expressed. In an RNA sequencing experiment, a potential selection for background genes could be all genes that are annotated to the reference genome, but more strict definitions are also reasonable. Their inclusion improves statistical accuracy. To allow quick reproduction and recapitulation of results from previously analyzed datasets, analyzed gene/protein lists, associated sets of background genes and user-selected parameter settings can be automatically re-uploaded into the application. Every time a dataset is submitted to analysis, the application will write related files into the result subdirectory “Input_data.” Automatic reimport is enabled after specification of that directory as the data source and selection of the “MBCO” default column names.

During any data upload, the application will always search for the parameter settings file in the specified input data directory. The user can copy the parameter settings file from the “Input_data” directory into a directory that contains new data to reuse selected parameters on that dataset or for external modification of parameter settings.

If “Custom 1” or “Custom 2” column name sets are selected, the application will save the user-specified names after data upload into the ‘Datasets_generated_by_app’ directory to allow their automatic reimport into the application after restart. Similarly, the application will save which default column names were selected and if one or all files in a directory were imported to restore the last selections after restart.

### 2.5 Manual upload and assignment of background genes

Besides using the functionalities of the “Read data”-menu, upload and mapping of background genes can additionally be achieved using the menu “Background genes.”

### 2.6 Definition of significance cutoffs for user-supplied data

The next menu, “Set data cutoffs”, allows for definition of significance cutoffs that determine which genes of each dataset will be subjected to enrichment analysis.

### 2.7 Organization of user-supplied data

One of the main functions offered by the “Organize data” menu is the quick assignment of integration groups and colors to the user-supplied datasets as well as their deletion. This is enabled by temporal grouping of datasets based on selectable shared characteristics. Described actions will be applied to all datasets within the same temporal group. Integration groups and colors can automatically be assigned. Among the selectable characteristics are shared time points, directionalities of change (e.g., up- or downregulation) or shared user-selected substrings within the dataset names. Based on a user-supplied delimiter, the application will internally split the dataset names into multiple substrings. For example the name “daunorubicin - healthy hiPSCd cardiomyocyte line MSN09 - outlier response” will be split into “daunorubicin,” “healthy hiPSCd cardiomyocyte line MSN09” and “outlier response,” if “ - ” was selected as a delimiter. The user can then specify the position index of the relevant substring, counted from the left and/or the right. For definition of multiple substrings whose combined matching will define a temporal group the user can separate the indices by commas. As another functionality, the menu “Organize data” allows the specification of the order of the results for datasets of the same integration group in bar diagrams, heatmaps and pie charts.

### 2.8 Selection of an ontology and the species under investigation

Within the “Ontology/Species” menu the user can select an ontology of interest and the species under investigation. If existent, the application will upload a species-selective version of the selected ontology. Otherwise, it will replace human genes by their species orthologues, using the Mouse Genome Informatics and NCBI orthologue databases (Sayers *et al.* 2019, Blake *et al.* 2021) as described previously (Hansen *et al.* 2024). The user will be asked to download any missing files, as described in the related supplied text files. Since other ontologies can be continuously updated, we recommend to document the download date. For investigation of the selected ontology, the menu allows writing of parent child networks that can be opened with the graph editor specified in the “SCP networks” menu. In the case of MBCO, this menu additionally enables writing of networks showing the inferred level-2 and -3 SCP weighted interactions. The user can specify the percentage of top interactions to be visualized for each level.

### 2.9 Definition of enrichment parameters and graphical outputs

The menu “Enrichment” allows specification of enrichment parameters and selection of types of figures to be generated for display of results. User-supplied datasets are subjected to standard enrichment analysis that is based on the right-tailed Fisher’s exact test. This menu allows definition of two SCP significance cutoffs, i.e. maximum significance *P* value and rank. Pathways are significant, if they meet both cutoffs. Since MBCO SCPs are in parent and child relationships across the four different levels but show minimal redundancies between SCPs within the same level, the application enables the definition of cutoffs for each SCP level separately. In addition, MBCO allows dynamic enrichment analysis. Briefly, dynamic enrichment analysis merges functionally related SCPs that contain at least one gene of the analyzed dataset into dataset-selective SCP combinations and adds those to a dataset-selective ontology, before enrichment analysis using Fisher’s exact test. SCP cutoffs for dynamic enrichment analysis can be separately specified. Functional SCP relationships are defined by the weighted inferred MBCO SCP interactions and the user can select the percentage of the top interactions to be considered. In the case of the three GO namespaces, i.e. biological processes, cellular components and molecular functions, the “Enrichment” menu allows removal of too large or too small GO terms by specification of a maximum and minimum number of genes within a considered GO term.

### 2.10 Definition of SCP network-related features

The “SCP-networks” menu allows users to specify the criteria for connecting SCP nodes, define the visualization rules for SCP nodes and pie charts, and choose the graph editor for network visualization.

The user can select if predicted SCPs of the same level shall be boxed and if SCPs should be connected across all datasets of the same integration group based on parent-child and/or functional relationships. Percentages of considered top inferred MBCO interactions can be specified for standard enrichment analysis, while they are the same as those specified in the menu “Enrichment” for dynamic enrichment analysis.

Genes with altered expression that map to predicted pathways can be added to the networks as child nodes.

Since the hierarchical organization of other ontologies often contains parent-child relationships over multiple levels, the user can specify, if predicted pathways shall only be connected by intermediate nodes or if all of their ancestors should be added. In the case of GO, only ancestors are considered that fulfill the size requirements specified in the menu “Enrichment.” Additionally, GO pathways can be connected based on regulatory interactions.

SCP nodes will be visualized as pie charts where each slice represents one dataset that predicted that SCP meeting the significance criteria specified in the menu “Enrichment.” The user can select the slice and pie chart areas to be proportional to each −log_10_(*P* value) and the sum of all −log_10_(p-values) predicted for related datasets, respectively. −log_10_(*P* values) predicted by dynamic enrichment analysis will be split equally among all contributing SCPs and multiple split −log_10_(*P* values) for the same SCP will be summed up. For further emphasis on SCPs predicted with high significance and by many datasets, the application allows selecting that SCP label sizes increase with increasing SCP node areas. Label size ranges can be specified. As alternative approaches for visualization of results in pie charts, pie chart areas can be selected to be proportional to the total number of related datasets or the number of related datasets assigned to the same color. Same color assignments can, for example, be used to highlight the high-throughput assays, if each generated multiple datasets [e.g. to compare sc and sn transcriptomics, if each assay predicted multiple subtypes for a cell type of interest (Hansen *et al.* 2022b)]. As a last option, the user can select that pie charts have identical sizes for all SCPs. In case of the three last discussed selections slice sizes will be identical within the same pie chart. Node scaling can be set to be unique for each network or to be the same for all networks predicted by standard or dynamic enrichment analysis. The user can either select the yED graph editor (yWorks GmbH 2025) or Cytoscape (Shannon *et al.* 2003) for network visualization.

### 2.11 Selection of SCPs of interest for focused analysis

The menu “Select SCPs” allows grouping of SCPs or hierarchical SCP branches that will be shown in all result figures and networks, if the check box at the bottom of the menu is activated. Results figures or networks will be generated for all SCPs of the same group, while significance cutoffs will be ignored. To analyze which dataset genes map to selected SCPs, dataset genes can be added as child nodes to the SCPs.

### 2.12 Definition of additional SCPs

Within the menu “Define own SCPs” the user can generate new SCPs by merging existing SCPs, which will be added to the ontology.

### 2.13 Data analysis

Pressing the “Analyze” button will subject all uploaded datasets to standard and, if MBCO is selected, dynamic enrichment analysis. Bardiagrams, heatmaps, and timelines will be generated, if selected in the “Enrichment” menu and saved in the specified results directory. Additionally, they will be prepared for visualization within the application, if not deactivated in the “Results” menu. Networks will be generated for the yED or Cytoscape editor, as selected in the “SCP networks” menu and saved in the specified results directory. The application will give a brief instruction how to proceed. Furthermore, the application writes a read me file for the use of the yED graph editor or Cytoscape into the directory that also contains the network files.

### 2.14 Bundling of data upload and analysis

To reduce manual steps, the button “Clear, read & analyze” will delete all uploaded datasets and background gene lists, upload user-supplied data using the instructions specified in the “Read data” menu and immediately subject the data to enrichment analysis. Since - as described above - the application will reupload the last selections within the “Read data” menu after restart and any specified parameter settings will be automatically imported from the selected data directory, this functionality can allow quick data processing without additional manual steps.

### 2.15 Automatic analysis using command line code

To bypass all manual interactions with the user interface, a complete analysis cycle containing data upload, parameter specification, and analysis can be started from the command line. The user can specify a few arguments including the directory of the input data. The application will import all data files, files with background genes, the parameter settings and if specified, the custom column name sets (that map data columns to the data fields in the application) from that directory. Data will automatically be subjected to enrichment analysis. In case of error-free performance, the application will close and write “Analysis finished” text files into multiple directories that can be used as a signal by enwrapping programs. If any errors (e.g. during data upload) are encountered, the user interface will become visible and be kept open to allow error investigation.

We provide a command line automation guide file for instructions and an R-script to launch MBC PathNet within R.

### 2.16 Instruction for the reproduction of the figures

For the reproduction of the figures shown in this manuscript, first download all or at least the Gene Ontology related datasets using the Windows or Linux commands given in the related instruction files in the main folder of the application. Additionally download the yED graph editor (or alternatively Cytoscape), as instructed in the same files.

**Figure 1.**
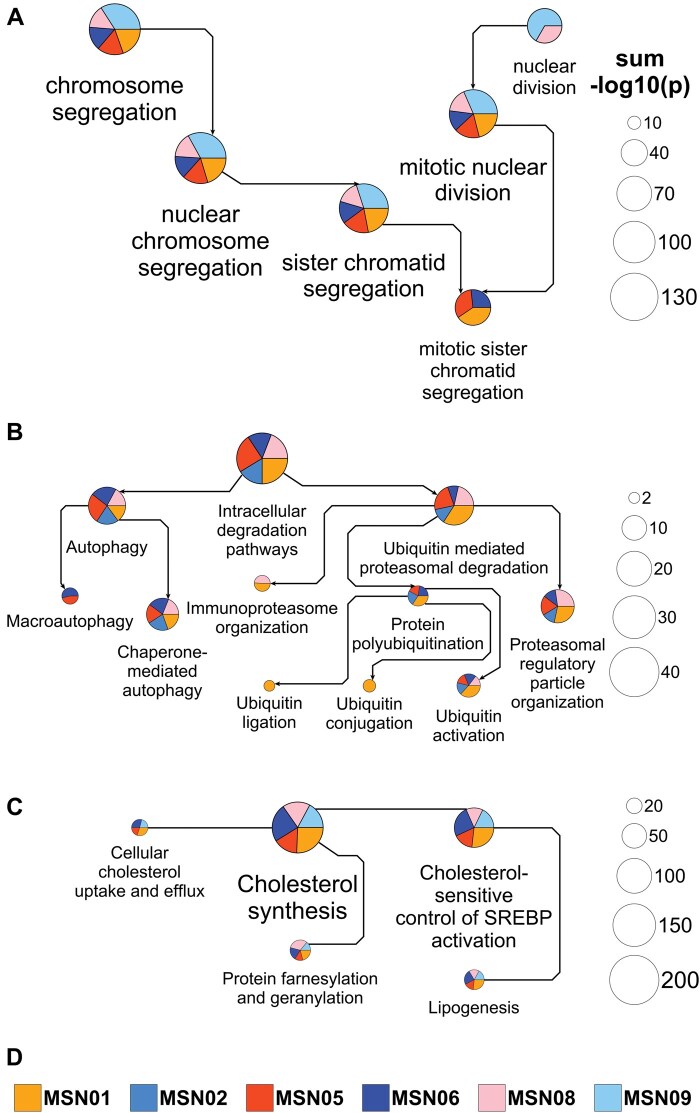
Pathway networks generated from drug-induced up- or downregulated genes. Up- or downregulated genes among the top 600 differentially expressed genes induced by three example drugs in up to six healthy human subject iPSC-derived cardiomyocyte cell lines were subjected to pathway enrichment analysis using MBC PathNet. (A) The top five downregulated GO Biological Processes (min/max # pathway genes: 5/360) for each of five out of six treated cell lines were integrated into the GO directed acyclic graph. Results obtained for the sixth cell line describe extracellular matrix processes and are excluded from the figure. Pie chart slice and total areas are proportional to individual and the sum of all shown –log_10_(*P* values), respectively, documenting pathway significance levels in the color-coded cell lines (see legend in D). Arrows point from parent to child processes. Process labels were selected to increase and decrease with pie chart sizes. Please note that for this analysis we used a GO version downloaded on 24 May 2025. The continuous update of GO can challenge the reproducibility of the shown results. (B) The top 5, 5, 10, and 5 level-1, -2, -3, and -4 MBCO SCPs upregulated by bortezomib in five cell lines were integrated into the MBCO parent-child hierarchy. Enrichment results are shown for the SCP “Intracellular degradation pathways” and all its descendants. Each arrow points from a parent to its child SCP, allowing identification of SCP levels by counting the arrows that separate an SCP from the overall level-1 parent. Note that we selected unique SCP label sizes for this visualization. (C) Enrichment analysis of lapatinib-upregulated genes in five cell lines using MBCO and dynamic enrichment analysis predicts a level-3 SCP-network focusing almost exclusively on cholesterol-related SCPs (generated from the top five predictions). SCPs are connected, if they belong to the top 25% inferred interactions between level-3 SCPs that were also used as the network basis for dynamic enrichment analysis. (D) Pie slice colors in A-C indicate the cell line in which the corresponding pathway was significantly induced.

To generate Fig. 1A, start the application and open the “Example data” menu and load the “LINCS DToxS/predicTox examples” dataset. Then open the “Ontology/Species” menu and select “GO biological process” in the “Ontology” list box at the top of the application. The application will search for the necessary data files and document if any necessary files are missing. If generated pathway networks shall be visualized with Cytoscape, change the graph editor at the bottom of the “SCP networks” menu. For faster network generation, you can uncheck “Bardiagrams” in the “Enrichment” menu. Now press the “Analyze” button.

In case of first time use of GO, the application will populate all GO terms with the genes of their child terms, based on the “is_a” and “part_of” relationships. It will additionally generate a file that contains only those GO terms that meet the minimum and maximum size requirements that are specified in the “Enrichment” menu. Following these steps the application will start the enrichment analysis, generate the charts selected in the “Enrichment” menu and the pathway networks. Open the “SCP networks” subdirectory in the “Results/LINCS_DToxS_predicTox” folder, as also instructed by the application. The file “Go_bp_human_prednisolone-Down_standard” with the extension “.graphml” or “.xgmml” contains the visualized network in Fig. 1A, if yED graph editor or Cytoscape was selected, respectively. If yED is selected as the graph editor, the network can be opened with yED simply by selecting the network file. Within yED, select “Tree” - “Directed” in the “Layout” menu and activate “Consider Node Labels” in the “Directed” tab in the appearing pop-up window. SCP nodes can also manually be rearranged. Finally, select “Edge routing” - “Orthogonal/Polyline” in the “Layout” menu. Delete the network results for the cell line MSN02 that we identified as a cell line with an outlier response (Hansen *et al.* 2024). The network can be exported from yED into a PDF, using the “Export” command in the “File” menu.

Please note that GO is continuously updated, challenging the reproducibility of the presented results. This can affect *P* values as well as which pathways will pass the selected minimum and maximum size requirements (here, 5 and 360, respectively).

To generate Fig. 1B, select “MBCO” in the “Ontology/Species” menu and deactivate “Label sizes ∼ Node sizes” in the “SCP networks” menu. Press the “Analyze” button to start the second analysis. Generated SCP networks for standard (and dynamic) enrichment analysis of bortezomib-upregulated genes can be processed with yED (or Cytoscape, if selected), as described above.

For the generation of Fig. 1C, reactive “Label sizes ∼ Node sizes” within the “SCP networks” menu of the application. Set the rank cutoff level-2 SCP predictions obtained by dynamic enrichment analysis to 0 in the “Enrichment” menu to ensure that the legend covers only level-3 predictions. Press the “Analyze” button for a third time. SCP networks for dynamic enrichment analysis of lapatinib-upregulated genes can be prepared as described above.

## 3 Results

MBC PathNet can identify pathways networks underlying whole cell function, e.g. transport or proliferation, from bulk and single cell transcriptomic and proteomic datasets. We have used the functionalities implemented in MBC PathNet to integrate multiomics datasets obtained from undiseased human reference kidney samples within the Kidney Precision Medicine Project (KPMP) (Hansen *et al.* 2022b), for the documentation of SCP networks triggering neurite outgrowth (Hansen *et al.* 2022a) and the description of potential mechanisms associated with cardiotoxic tyrosine kinase inhibitors (Hansen *et al.* 2024) within the LINCS program.

Using the cardiotoxicity study, we here reanalyzed the gene expression profiles (Hansen *et al.* 2024, 2025) obtained for prednisolone, bortezomib, and lapatinib in six healthy human subject iPSC-derived cardiomyocytes. Enrichment analysis of genes downregulated by prednisolone using GO Biological Processes (that we downloaded on 24 May 2025) aligns with prednisolone’s inhibitory effect on cell cycle progression (Mattern *et al.* 2007) (Fig. 1A). Our application projected the top five predicted GO biological processes with a minimum and maximum number of five and 360 annotated genes into the GO directed acyclic graph to document parent-child (and regulatory) GO relationships. Excluding the top processes predicted for the cell line MSN02 (not shown), all processes describe mitotic chromosome and nuclear division events, aligning with prednisolone’s anti-proliferative effect. Genes upregulated by the proteasome inhibitor bortezomib were resubmitted to standard enrichment analysis using MBCO. Building on our first analysis (Hansen *et al.* 2024), we here integrated the top 5, 5, 10, and 5 level-1, -2, -3, and -4 SCPs into the MBCO hierarchy of parent-child relationships. Many of the SCPs predicted with high significance mapped to the “Intracellular degradation pathways” branch, suggesting a compensatory response to overcome proteasomal inhibition (Fig. 1B). Level-3 SCP networks generated by dynamic enrichment analysis (using the top five predictions) contained almost exclusively SCPs related to proteasomal degradation and autophagy (not shown), further supporting this hypothesis. Genes upregulated by lapatinib were subjected to dynamic enrichment analysis using MBCO. Top five level-3 predictions identify lapatinib’s influence on cholesterol homeostasis to an even greater extent than our initial results obtained by standard enrichment analysis (Hansen *et al.* 2024). In agreement, lapatinib-resistance has been linked to upregulated mevalonate pathway activities in cultured breast cancer cells that could be reversed by statin treatment (Sethunath *et al.* 2019).

In summary, MBC PathNet allows quick and easy upload, analysis and integration of multiple omics datasets. Generated SCP networks help with fast and simultaneous investigation and interpretation of the enrichment results for multiple datasets to identify the cell level processes that are affected by the experimental condition.

## Data Availability

Data used for all analyses can be found in the “Custom data” folder within the main directory of MBC PathNet.
